# Normal erythrocyte calpain I activity on membrane proteins under near-physiological conditions in patients with essential hypertension

**DOI:** 10.1590/S1516-31802002000100002

**Published:** 2002-01-02

**Authors:** Tereza Maria Dantas de Medeiros, Katia Coelho Ortega, Décio Mion, Kimiyo Nonoyama, Orlando Cesar de Oliveira Barretto

**Keywords:** Calpain I, Calpastatin, Red cell membrane proteins, Band 2.1, Band 4.1, Essential hypertension, Calpaina I, Calpastatina, Proteinas da membrana eritrocitaria, Banda 2.1, Banda 4.1, Hipertensão essencial

## Abstract

**CONTEXT:**

It has been reported that the equilibrium between the erythrocyte protease calpain I and its physiological inhibitor calpastatin is disrupted in patients with essential hypertension.

**OBJECTIVE:**

To investigate the activity of non-purified calpain lin hemolysates against the erythrocytic membrane proteins, rather than against other substrates.

**DESIGN:**

Evaluation of calpain I red cell activity upon its own physiological substrates in hypertensive patients, in a near-physiological environment.

**SETTING:**

LIM-23 and LIM-40 of Hospital das Clinicas of the Faculty of Medicine of USP.

**SAMPLE:**

Patients with moderate primary hypertension over 21 years of age who were given amlodipine (n:10) and captopril (n:10) for 8 weeks, plus normal controls (n:10).

**MAIN MEASUREMENTS:**

Red cell membrane proteins were incubated with and without protease inhibitors and with and without calcium chloride and underwent polyacrylamide gel electrophoresis.

**RESULTS:**

Digestion of bands 2.1 and 4.1 was observed, indicating calpain I acitivity. No statistical differences regarding bands 2.1 and 4.1 were observed before treatment, between the controls and the hypertensive patients, either in ghosts prepared without calcium or with increasing concentrations of calcium. Nor were statistical differences observed after treatment, between the controls and the patients treated with amlodipine and captopril, or between the patients before and after treatment with both drugs.

**CONCLUSION:**

The final activity of non-purified calpain I upon its own physiological substrate, which was the approach utilized in this study, may more adequately reflect what happens in red cells. Under such conditions no imbalance favoring calpain I activity increase was observed. The protective factor provided by calpastatin against calpain I activity may diminish under hypertension.

## RESUMO

**CONTEXTO:**

A quebra do equilíbrio entre a protease erritrocitária calpaina I e o seu inibidor fisiológico calpastatina tem sido relatado em pacientes com hipertensão essencial.

**OBJETIVO:**

Neste trabalho a atividade da calpaina I presente nos hemolisados foi investigada contra as próprias proteinas da membrana eritrocitária

**TIPO DE ESTUDO:**

avaliação da atividade da calpaina I contra os próprios substratos fisiológicos em pacientes hipertensos em ambiente aproximandamente fisiológico.

**LOCAL:**

LIM-23 e LIM-40 do Hospital das Clinicas da Faculdade de Medicina da USP

**PACIENTES:**

Pacientes com hipertensão primária moderada que receberam amlodipina (n:10), captopril (n:10), bem como controles normais (n:10)

**VARIÁVEIS ESTUDADAS:**

Proteinas da membrana eritrocitária foram incubadas com e sem inibidor, e com e sem cloreto de calcio.

**RESULTADOS:**

As bandas 2.1 e 4.1 foram digeridas, indicando a atividade da calpaina I. Não foram encontradas diferenças estatisticamente significantes em relação às bandas 2.1 e 4.1 entre os controles e os pacientes antes do tratamento, seja sem calcio seja com concentrações crescentes de cálcio. Também não foram encontradas diferenças estatísticamente significantes entre os controles e os pacientes depois do tratamento com amlodipin e captopril, bem como entre os pacientes antes e depois do tratamento.

**CONCLUSÃO:**

A atividade final da calpaina I não purificada contra os seus próprios substratos fisiológicos pode refletir mais adequadamente o que acontece no glóbulo vermelho, e nessas condições, não foi encontrado desequilíbrio favorecendo o aumento da atividade da calpaina I. Fatores protetores da calpastatina à atividade da calpaina I existentes na hipertensão poderiam estar diminuidos.

## INTRODUCTION

The cause of essential hypertension remains unknown, but it has been accepted that hypertensive patients present an increased genetic sensitivity to a series of environmental influences.^1^ It is known that some specific red blood cell membrane functions are abnormal in hypertensive patients, such as abnormal net Na^+^, K^+^ and Ca^2+^ fluxes in erythrocyte membranes,^2-5^ as well as in rats with spontaneous hyper-tension.^6^ Hypertensive disease is accompanied by an abnormal increase in Ca^2+^ in many cells, and Ca^2+^ channel blockers are used to treat hypertension.^5^ This dysfunction may be related to proteolysis of proteins involved in ion transportation. In fact, it has been reported that the equilibrium between the endogenous red cell protease calpain I and its physiological inhibitor calpastatin is disrupted in hypertensive patients, with an imbalance of the proteolytic system occurring, possibly due to environmental stimuli.^7-10^ This disequilibrium would lead to structural membrane disturbances, increased free intracellular calcium and a disturbance of the cellular proteolytic system,^8-10^ Such an imbalance would affect some red cell membrane proteins and the cation transportation system, as Na^+^-K^+^ co-transportation increases and leads to decreased intracellular Na+ followed by hypertension.^7^

These studies have been carried out with erythrocytes, as these cells have been successfully used as a mirror for what happens in other tissues, for example lymphocyte adenosine deaminase deficiency, which may be assessed in the red cells.

It is known that calpain I (EC 3.4.22.17) is a red cell protease which acts selectively upon the erythrocyte membrane proteins 2.1 and 4.1, and is activated by 0.05 mM calcium.^11,12^ Its effect in vivo is modulated by its specific physiological inhibitor, calpastatin.^13^

All the cited reports dealt with purified enzyme preparations and purified non-membrane substrates like hemoglobin. Natural substrates like membrane proteins have not been employed. That being so, this study was designed in order to observe the action of non-purified enzymes in a near-physiological environment upon their own membrane proteins as the physiological substrate.

## METHODS

In this work, calpain I activity on the erythrocyte membrane proteins of normal controls (n:10) was investigated, as well as on primary hypertension patients over 21 years of age, with mild to moderate hypertension, as defined according to the V Joint National Committee.^14^ Informed consent was given by all subjects. The patients were given the calcium channel-blocker amlodipine (n:10) and the angiotensin-converting enzyme-blocker captopril (n:10) for 8 weeks. A two-week washout period was instituted before treatment. Venous blood samples from normal controls and hypertensive patients were taken for membrane studies, before and after the 8 weeks of treatment. The membrane preparations were obtained from erythrocytes that were cleared of leukocyte contamination using 1:1 microgranular cellulose to alpha cellulose column filtration, in order to avoid the presence of the white blood cell protease, which could jeopardize the results.

Afterwards, the red cells were washed in buffered saline pH 7.4 at 4°C and lysed 1:20 in 5 mM phosphate buffer pH 7.4 without any protease inhibitor. Thereafter, the hemolysates with ghosts in suspension, containing both crude enzymes and crude substrates, were divided into aliquots and incubated at 37°C with 0.05 mM, 1.0 mM and 2.0 mM calcium chloride concen-trations, all with 0.18 mM chloramphenicol for 1 hour at 37°C.

The ghosts were prepared according to Dodge et al.^15^ by washing them six times in 5 mM phosphate buffer pH 7.4 at 19,000g at 4°C. The ghosts were solubilized and applied in SDS-PAGE at 10% linear acrylamide (80 mg of ghost in each well), according to Laemmli,^16^ and at 3-17% exponential acrylamide gradient (100 mg of ghost in each well), according to Fairbanks.^17^ For every patient as well as for every control, a ghost without calcium was also prepared with 0.2 mM phenylmethylsulfonyl fluoride (PMSF) as a serine protease inhibitor, and 2 mM nethylmaleimide (NEM) as a cysteine protease inhibitor. In order to check whether calpain I was present, hemolysates from normal controls were incubated for 1 hour at 37°C under the following conditions: 1) hemolysate without calcium and without inhibitor; 2) hemolysate + 2.0 mM calcium without inhibitor; 3) hemolysate + 2.0 mM calcium + 0.2 mM PMSF; 4) hemolysate + 2.0 mM calcium + 2.0 mM NEM.

The gels were stained with coomassie blue and scanned in a LKB Ultroscan XL Laser densitometer with LKB gelscan/92 software. The band concentrations were given as the percentage of the total red cell membrane protein. Spectrin and ankyrin concentrations were determined in the Fairbanks gels; band 3 and other small membrane proteins were determined in the Laemmli gels.

Variance analysis, as well as the Tukey-Kramer multiple comparison, was used for detecting any statistical difference at 5% significance level among the studied groups.

## RESULTS

**Figure 1:**
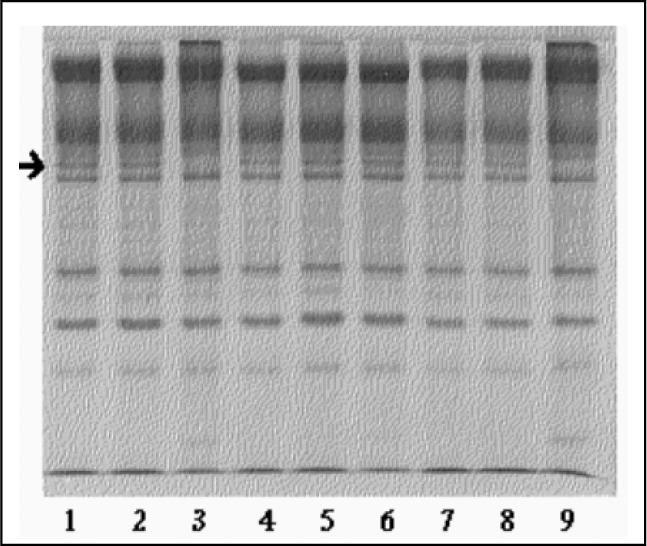
Linear polyacrylamide gel electrophoresis (SDS-PAGE) of red cell membrane proteins for controls. Hemolysates prepared with buffer with and without inhibitor, with or without calcium, were incubated (shown below) for 1 hour at 37 °C. The arrow points at band 4.1.

**Figure 2:**
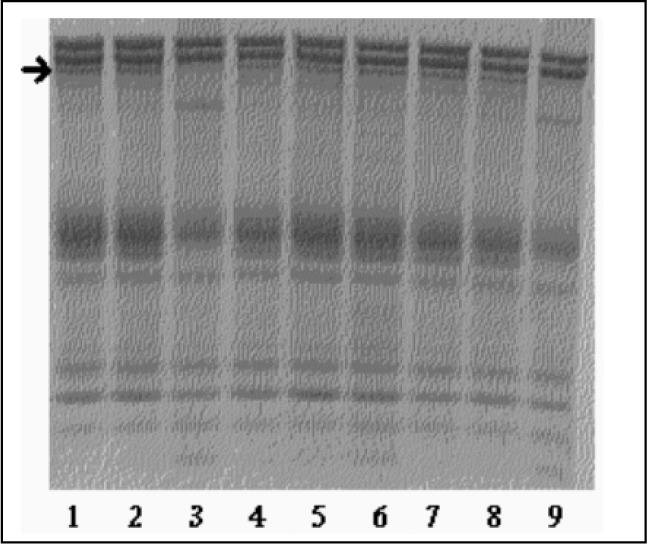
Gradient polyacrylamide gel electrophoresis (SDS-PAGE) of red cell membrane proteins for controls. Hemolysates prepared with buffer with and without inhibitor, with or without calcium, were incubated (shown below) for 1 hour at 37 °C. The arrow points at band 2.1.

The first experiments confirmed that the hemolysates presented a protease that is active with calcium on bands 2.1 and 4.1, is not inhibited by PMSF (serine protease inhibitor) and is inhibited by NEM (cysteine protease inhibitor). This indicated the presence of calpain I, the only described red cell cysteine protease presenting these features. Figures 1 and 2 clearly show the disappearance of bands 2.1 and 4.1 in lanes 3 and 9. In lane 3, the calcium led to proteolysis and in lane 9, the PMSF did not inhibit the proteolysis, confirming that this proteolysis could be ascribed to calpain I activity. Moreover, in the lanes that had NEM, a calpain I inhibitor, no proteolysis was observed. According to our data, laid out in Tables 1 and 2, bands 2.1 and 4.1 were digested, indicating calpain I activity. Band 2.1 started to be degraded with 0.05 mM calcium chloride, whereas band 4.1 degradation was only achieved with 2 mM calcium. Whenever a band showed proteolysis, other minor bands started to appear, indicating that these ones might be degradation products (Figure 1).

**1. hemolysate + no Ca^2+^, no inhibitor, not incubated; 2. hemolysate + no Ca^2+^, no inhibitor, incubated at 37 °C for 1 hour; 3. hemolysate + 2.0 mM Ca^2+^, no inhibitor, incubated at 37 °C for 1 hour; 4. hemolysate + no Ca^2+^, 2.0 mM NEM, not incubated; 5. hemolysate + no Ca^2+^, 2.0 mM NEM, incubated at 37 °C for 1 hour; 6. hemolysate + 2.0 mM Ca^2+^, 2.0 mM NEM, incubated at 37° C for 1 hour; 7. hemolysate + no Ca^2+^, 0.2 mM PMSF, not incubated; 8. hemolysate + no Ca^2+^, 0.2 mM PMSF, incubated at 37 °C for 1 hour; 9.hemolysate + 2.0 mM Ca^2+^, 0.2 mM PMSF, incubated at 37 °C for 1 hour**

**1. hemolysate + no Ca^2+^, no inhibitor, not incubated; 2. hemolysate + no Ca^2+^, no inhibitor, incubated at 37 °C for 1 hour; 3. hemolysate + 2.0 mM Ca^2+^, no inhibitor, incubated at 37 °C for 1 hour; 4. hemolysate + no Ca^2+^, 2.0 mM NEM, not incubated; 5. hemolysate + no Ca^2+^, 2.0 mM NEM, incubated at 37 °C for 1 hour; 6. hemolysate + 2.0 mM Ca^2+^, 2.0 mM NEM, incubated at 37 °C for 1 hour; 7. hemolysate + no Ca^2+^, 0.2 mM PMSF, not incubated; 8. hemolysate + no Ca^2+^, 0.2 mM PMSF, incubated at 37 °C for 1 hour; 9. hemolysate + 2.0 mM Ca^2+^, 0.2 mM PMSF, incubated at 37 °C for 1 hour.**

**Table 1:** Percentage of total red cell membrane proteins in bands 2.1 and 4.1 for controls and patients treated with captopril and amlodipine - before the treatment.

	*Control* *n:10*	*Captopril* *n:10*	*Amlodipine* *n:10*	*P value* *(**1**)* *Anova*
***Band 2.1***				
*No calcium*	*6.5 ± 0.4*	*6.1 ± 0.4*	*6.0 ± 0.6*	*0.15*
*0.05 mM Ca^2+^*	4.9 ± 0.7*	4.3 ± 0.5*	4.6 ± 0.4*	*0.33*
*1.0 mM Ca^2+^*	3.5 ± 1.0*	3.8 ± 0.8*	3.7 ± 0.5*	*0.81*
*2.0 mM Ca^2+^*	2.1 ± 0.3*	2.5 ± 0.3*	2.9 ± 0.7*	*0.09*
*Anova - p (* *2* *)*	*0.01 - 0.001*	*0.001*	*0.001*	
***Band 4.1***				
*No calcium*	*7.1 ± 0.6*	*7.0 ± 0.7*	*6.7 ± 0.6*	*0.56*
*0.05 mM Ca^2+^*	*6.7 ± 0.8*	*6.8 ± 0.6*	*6.7 ± 0.3*	*0.95*
*1.0 mM Ca^2+^*	*5.8 ± 0.8*	*6.5 ± 1.0*	*6.3 ± 0.6*	*0.46*
*2.0 mM Ca^2+^*	4.9 ± 0.9*	4.7 ± 1.4*	5.4 ± 0.7*	*0.52*
*Anova - p (2)*	*0.01*	*0.01*	*0.01*	

(^1^) *Anova test between controls and captopril and amlodipine-treated patients*

(^2^) *Anova test among remaining levels of bands 2.1 and 4.1 with increasing calcium concentration (columns).*

(^*^) *Band 2.1 degradation started to be observed at 0.05 mM calcium and band 4.1 degradation was achieved at 2.0 mM calcium*

**Table 2:** Percentages of total red cell membrane proteins in bands 2.1 and 4.1 for controls and patients treated with captopril and amlodipine - after the treatment

	*Control* *n:10*	*Captopril* *n:10*	*Amlodipine* *n:10*	*P value* *(**1**)* *Anova*
***Band 2.1***				
*No calcium*	*6.5 ± 0.4*	*6.2 ± 0.4*	*6.0 ± 0.3*	*0.06*
*0.05 mM Ca^2+^*	4.9 ± 0.7*	4.0 ± 0.7*	4.1 ± 0.4*	*0.10*
*1.0 mM Ca^2+^*	3.5 ± 1.0*	3.3 ± 0.8*	3.9 ± 0.6*	*0.46*
*2.0 mM Ca^2+^*	2.1 ± 0.3*	2.5 ± 1.2*	2.6 ± 0.4*	*0.54*
*Anova - p (2)*	*0.01 - 0.001*	*0.01 - 0.001*	*0.001*	
***Band 4.1***				
*No calcium*	*7.1 ± 0.6*	*7.3 ± 1.0*	*7.1 ± 0.3*	*0.73*
*0.05 mM Ca^2+^*	*6.7 ± 0.8*	*6.5 ± 0.5*	*6.8 ± 1.2*	*0.82*
*1.0 mM Ca^2+^*	*5.8 ± 0.8*	*6.5 ± 0.4*	*6.7 ± 1.2*	*0.34*
*2.0 mM Ca^2+^*	4.9 ± 0.9*	5.3 ± 0.5*	4.9 ± 0.6*	*0.52*
*Anova - p (2)*	*0.01*	*0.001*	*0.01*	

(^1^) *Anova test among control, captopril and amlodipin treated patient*

(^2^) *Anova test among remaining band 2.1 and band 4.1 levels with increasing calcium concentration (columns).*

(^*^) *Band 2.1 degradation began to be observed at 0.05 mM calcium and band 4.1 degradation was achieved at 2.0 mM calcium.*

No statistical differences regarding band 2.1 and band 4.1 were observed before treatment, between the controls and the hyper-tensive patients, either in ghosts prepared without calcium or with increasing concentrations of calcium (Table 1). Nor were statistical differences observed after treatment, between the controls and the patients treated with amlodipine and captopril (Table 2), or between the patients before and after treatment with both drugs (Tables 3 and 4).

**Table 3:** Percentages of total red cell membrane proteins in bands 2.1 and 4.1 for the patients treated with captopril - before and after the treatment

	*Control* *n:10*	*Amlodipin before treatment* *n:10*	*Amlodipin after treatment* *n:10*	*P value* *Anova*
***Band 2.1***				
*No calcium*	*6.5 ± 0.4*	*6.1 ± 0.4*	*6.2 ± 0.4*	*0.19*
*0.05 mM Ca^2+^*	*4.9 ± 0.7*	*4.3 ± 0.5*	*4.0 ± 0.7*	*0.20*
*1.0 mM Ca^2+^*	*3.5 ± 1.0*	*3.8 ± 0.8*	*3.3 ± 0.8*	*0.60*
*2.0 mM Ca^2+^*	*2.1 ± 0.3*	*2.5 ± 0.3*	*2.5 ± 1.2*	*0.63*
***Band 4.1***				
*No calcium*	*7.1 ± 0.6*	*7.0 ± 0.7*	*7.3 ± 1.0*	*0.71*
*0.05 mM Ca^2+^*	*6.7 ± 0.8*	*6.8 ± 0.6*	*6.5 ± 0.5*	*0.72*
*1.0 mM Ca^2+^*	*5.8 ± 0.8*	*6.5 ± 1.0*	*6.5 ± 0.4*	*0.36*
*2.0 mM Ca^2+^*	*4.9 ± 0.9*	*4.7 ± 1.4*	*5.3 ± 0.5*	*0.59*

**Table 4:** Percentages of total red cell membrane proteins in bands 2.1 and 4.1 for the patients treated with amlodipine - before and after the treatment

	*Control* *n:10*	*Amlodipin before treatment* *n:10*	*Amlodipin after treatment* *n:10*	*P value* *Anova*
***Band 2.1***				
*No calcium*	*6.5 ± 0.4*	*6.0 ± 0.6*	*6.0 ± 0.3*	*0.08*
*0.05 mM Ca^2+^*	*4.9 ± 0.7*	*4.6 ± 0.4*	*4.1 ± 0.4*	*0.06*
*1.0 mM Ca^2+^*	*3.5 ± 1.0*	*3.7 ± 0.5*	*3.9 ± 0.6*	*0.73*
*2.0 mM Ca^2+^*	*2.1 ± 0.3*	*2.9 ± 0.7*	*2.6 ± 0.4*	*0.08*
***Band 4.1***				
*No calcium*	*7.1 ± 0.6*	*6.7 ± 0.6*	*7.1 ± 0.3*	*0.32*
*0.05 mM Ca^2+^*	*6.7 ± 0.8*	*6.7 ± 0.3*	*6.8 ± 1.2*	*0.94*
*1.0 mM Ca^2+^*	*5.8 ± 0.8*	*6.3 ± 0.6*	*6.7 ± 1.2*	*0.35*
*2.0 mM Ca^2+^*	*4.9 ± 0.9*	*5.4 ± 0.7*	*4.9 ± 0.6*	*0.45*

## DISCUSSION

Red cells have been used as a tool for detecting other metabolic defects in tissue, and the Pontremoli group has reported decreased calpastatin in red cells in hypertensive patients.^8-10^ This would lead to a calpain I -calpastatin disequilibrium favoring calpain I activity, due to the decrease in calpastatin. That group purified calpain I as well as calpastatin, and observed a decrease in calpastatin concentration, which was ascribed to digestion of its own calpastatin by the calpain I.^9,10^

Enzyme activity inhibition or stimulation is usually achieved by modulators. Calpain I modulators have not been reported, except for calcium, and some reports^18,19^ have claimed that there is an increase in erythrocyte calcium in hypertensive patients as well, which could activate calpain I.

Although it has been reported that red cell calpastatin is decreased in hypertensive patients,^11-13^ which would possibly lead to higher final calpain I activity, this hypothesis was not confirmed under the conditions presented in this study. Here, the final calpain/calpastatin system activity did not increase the calpain I proteolytic effect upon bands 2.1 and 4.1 (Table 1).

It may be suggested that putative protective factors provided by calpastatin could be decreased in hypertension, and so calpastatin would be degraded. However, even a diminished concentration of calpastatin would be enough to inhibit calpain I activity in vivo, at least in the red cells.

Hypertensive patients and controls were studied, and no statistical differences were found between them, regarding membrane protein concentrations (Tables 1 and 2). Equally, no differences were observed before and after treatment with amlodipine or captopril (Tables 3 and 4).

The strategy designed for this study, which utilized crude hemolysate containing both crude enzymes and red cell membrane proteins as substrates, may more adequately reflect what actually happens in red cells. This procedure maintained an approximately physiological environment, in which other cytosol proteins or factors that are taken out during protein purification were not excluded. Under these conditions, no final increased activity of calpain I on its own membrane proteins was found in hypertensive patients. However, our results do not contradict the results cited from the literature, as these authors employed purified preparations.

Moreover, if an increase in calpain activity played a role in red cell physiology, it would lead to an increase in red cell hemolysis, as calpain I is very active upon the crucial protein bands 2.1 and 4.1 of the red cell cytoskeleton. Nevertheless, hemolysis enhancement is not found in hypertensive patients. These findings do not, however, rule out a role for the calpain I - calpastatin system in human renal cells. A report from Milan^20^ on kidneys from hypertensive rats has shown that proteases could act upon enzymes or other proteins involved in hypertension etiology.

In conclusion, this present study has not revealed any significant difference in the calpain I - calpastatin system in red cells, between hypertensive patients and normal controls. Protective factors provided by calpastatin against calpain I activity may be diminished in hypertension, which could explain why it has been reported that calpastatin concentration is decreased. However, this decrease in calpastatin concentration would be sufficient to inhibit calpain I activity, at least in the red cells.
